# “No One Truly Understands What We Go through and How to Treat It”: Lived Experiences with Medical Providers among Patients with Orofacial Pain

**DOI:** 10.3390/ijerph191610396

**Published:** 2022-08-20

**Authors:** Victoria A. Grunberg, Mira Reichman, Brenda C. Lovette, Ana-Maria Vranceanu, Jonathan Greenberg

**Affiliations:** 1 Center for Health Outcomes and Interdisciplinary Research, Department of Psychiatry, Massachusetts General Hospital/Harvard Medical School, Boston, MA 02114, USA; 2 Division of Newborn Medicine, MassGeneral for Children, Boston, MA 02114, USA; 3 Department of Psychology, University of Washington, Seattle, WA 98195, USA; 4 Department of Rehabilitation Sciences, MGH Institute of Health Professions, Boston, MA 02129, USA

**Keywords:** orofacial pain, patient–provider interactions, patient-centered communication

## Abstract

Orofacial pain affects 10–15% of adults, yet treatments are limited. The gaps in care are frustrating for both patients and providers and can negatively impact patient–provider interactions. These interactions are key because they impact patient-reported outcomes and satisfaction with care. Purpose: Our study aims to understand the nuanced experiences with medical providers among patients with orofacial pain. Methods: In a cross-sectional survey, 260 patients provided written responses describing their experiences with medical providers. Using an inductive–deductive approach to thematic analysis, we identified themes and subthemes and organized them into four domains based on the Patient-Centered Model of Communication. Results: Patients reported feeling hopeless about treatment options, frustrated with lack of provider knowledge, disappointed in ineffective care, and stigmatized and dismissed by providers. Patients also said they learned to advocate for their health, were grateful for effective care, and felt lucky when providers listened and showed compassion. Patients identified key barriers that interfere with care (e.g., insurance, transportation, limited providers, lack of team coordination). Conclusions: Findings can help inform training programs and psychoeducation that target patient–provider communication to improve patient-reported outcomes, the quality of care delivered, and health care utilization and costs.

## 1. Introduction

Chronic orofacial pain is an umbrella term for persistent pain (≥15 days in per month and lasting >3 months) in the face, mouth, or jaw [1,2], according to the International Classification of Orofacial Pain (ICOP). Orofacial pain impacts ~10–15% of adults [3] and diagnoses span across musculoskeletal (e.g., temporomandibular disorders), odontogenic (e.g., phantom tooth pain), neurovascular (e.g., facial migraine), and neuropathic (e.g., trigeminal neuralgia) pain [3,4] categories. Orofacial pain conditions are difficult to diagnose and treat [4] because of unclear etiology, pathology, and the interaction with psychosocial factors (e.g., depression, anxiety, coping, isolation) [5]. Given the complexity of orofacial pain, diagnosis and treatment often require multiple specialists, hospital visits, and procedures—which strain an overburdened healthcare system [6]. The limited knowledge and treatments for orofacial pain can be frustrating for both patients and providers and, in turn, negatively impact their interactions as well as patient and healthcare system outcomes.

Across primary care and dental settings, providers report feeling uncertain and ill-equipped to diagnose and treat orofacial pain [6]. A recent study indicated that among 100 patients with orofacial pain, on average, they were referred to 15 distinct specialties, received seven consultations, and 76% reported unsuccessful treatment outcomes [7]. Qualitative work indicates that patients who repeatedly seek care for orofacial pain perceive distrust, rejection, and skepticism from providers [8]. Similarly, providers often report that these patients are helpless and difficult [6]. Across medical populations, patient-provider interactions impact mutual trust, patient satisfaction, quality of care, and pain outcomes [9,10]. Because patients with orofacial pain tend to feel socially isolated [5], interactions with providers are pivotal for their well-being. Positive patient–provider interactions may help improve the quality of care delivered, patient-reported outcomes, patient satisfaction, and health care utilization and costs.

Patient-centered communication (PCC) is widely endorsed as necessary for high-quality, patient-centered care [11]. The Patient-Centered Model of Communication [11] is a theoretical and conceptual framework that outlines factors that can influence provider–patient interactions: (1) patient factors (e.g., illness severity); (2) provider factors (e.g., professional knowledge); (3) provider–patient relationship factors (e.g., trust); and (4) health system factors (e.g., access to care). Identifying factors linked to quality of patient–provider interactions is an important first step to inform psychoeducational and communication trainings for providers. Qualitative research is an optimal way to capture the nuanced factors relevant to these real-world interactions [12]. This approach can help capture individual perceptions and experiences, which is important given that patients with orofacial pain report feeling misunderstood [8]. No studies, to date, have used a theoretical model to qualitatively examines these patients’ experiences with providers.

## 2. The Current Study

The purpose of this study was to understand patients’ experiences with medical providers when they present with orofacial pain. Informed by the Patient-Centered Model of Communication [11], our aim was to identify patient, provider, relationship, and health system factors relevant to patient–provider interactions. A better understanding of these factors can help inform psychoeducational and communication trainings for providers. Ultimately, these trainings could help improve: (1) quality of care delivered to patients with orofacial pain; (2) patient isolation and stigmatization; (3) patient-reported outcomes and satisfaction with care; and (4) health care utilization, provider burden, and costs.

## 3. Methods

We conducted a qualitative secondary analysis using a large cross-sectional survey on adults’ experiences living with chronic orofacial pain. We analyzed data from an open-ended question asking participants about their experiences with medical providers. Massachusetts General Hospital Institutional Review Board approved all study procedures.

### 3.1. Participants

A total of 303 participants enrolled in this online cross-sectional survey study from March to June 2021. Of them, 260 completed the open-ended question relevant to this study. Inclusion criteria were as follows: (1) 18 years or older; (2) experienced nonmalignant facial pain (any type) for more than three months; (3) currently live in the U.S.; and (4) able to read and write English at 6th grade level (self-reported). Table 1 displays the socio-demographics of the study sample (*N* = 260). We classified self-reported diagnoses based on ICOP criteria [2]. Table 1 presents diagnostic categories (e.g., trigeminal neuralgia, other trigeminal neuropathic pain, classical trigeminal neuralgia with concomitant continuous pain, persistent idiopathic facial pain). Notably, 22% of participants endorsed multiple orofacial pain diagnoses. The average age of participants was 58.43 years old (SD = 26.16).

### 3.2. Procedures

We recruited participants through the newsletter of the Facial Pain Association (FPA), an organization that provides resources to individuals living with orofacial pain. Interested participants completed screening questions to determine eligibility. Those that met inclusion criteria provided a waiver of consent and completed measures through a secure web-based survey platform (REDCap) [13]. Here, we focus on participants’ responses to the following open-ended question: “What has been your experience with your medical providers about your facial pain thus far?” Participants provided written responses to this question in a text box with no character limits.

### 3.3. Data Analytic Plan

We used a hybrid inductive–deductive approach to thematic analysis [14]. We identified themes and subthemes directly from the data (inductive) and organized them within theory-informed domains from the Patient-Centered Model of Communication [11] (deductive). We used the four factors outlined in the Patient-Centered Model of Communication (i.e., patient factors, provider factors, provider–patient relationship factors, and health system factors) as a priori defined domains to organize our themes and subthemes.

First, members of the study team (VG, MR, BL, JG) independently coded 20% of the qualitative responses without an established framework (i.e., open coding) (*n* = 40). We met to discuss codes and organized them within a coding framework. Then, two coders (MR, BL) independently applied the coding framework to the rest of the data. We met as a team to refine the coding framework as needed and resolve discrepancies in coding. Based on the coded data, the full team collaboratively identified and refined emerging themes and subthemes. Finally, three members of the team (VG, MR, BL) organized themes and subthemes into the a priori defined domains. We allowed qualitative findings to overlap and contradict to reflect the complexity and interdependence of patient experiences [15,16].

## 4. Results

We identified 10 themes, which are organized within the domains informed by the Patient-Centered Model of Communication [11]. Table 2 displays all themes and subthemes in each domain. We describe the themes in detail below using illustrative quotes.

### 4.1. Domain 1: Patient Factors

#### 4.1.1. “It Was a Failure from the Start”: Hopeless and Avoidant about Seeking Care

Patients felt hopeless about treatment, which led to avoidance in seeking continued care. Because treatment options are limited for orofacial pain, patients felt lost and confused on how to find appropriate care. Many of them experienced failed treatments—making them feel that “there’s nothing left to try”. As one participant stated, “There is not one expert in my state. I’ve been told everything from ‘you don’t have a diagnosis’ to ‘just push past the pain’. I’ve been told I’m not bad enough to warrant further intervention. Also, that there’s nothing left to try. That this is the way it is for the rest of my life. It’s awful to feel there is nowhere to turn for help”. Patients who perceived limited knowledge across the medical community regarding orofacial pain tended to stop seeking care and/or avoided seeing providers for their pain.

#### 4.1.2. “I Needed to Take Control Myself”: Initiative and Agency Needed to Find Appropriate Care

Patients reflected that they needed to take agency over their treatment and health to find appropriate care. Given patients’ perceptions that providers had limited familiarity with orofacial pain and treatment options, they felt they needed to “educate medical providers” on their condition. Patients described the importance of doing their own research to be able to explain their condition to providers, advocate for their needs, and propose potential treatment approaches. As one participant noted, “We all have to educate ourselves, be responsible for trying to make the right decisions—learn, learn, and learn”.

### 4.2. Domain 2: Provider Factors

#### 4.2.1. “Most Don’t Have a Clue”: Frustrated with Lack of Knowledge about Condition and Treatment

Patients endorsed frustration related to providers’ limited knowledge on their condition. They said that many providers seemed “mystified” by their symptoms and seem to “grasp at straws trying to determine the cause”. Patients were often misdiagnosed and had to “bounce from one doc to the next” to receive an accurate diagnosis. Patients said it took a long time to receive the right diagnosis and/or treatment—if they were lucky enough to find effective treatment. As one participant said, “Every doctor I have seen over the past year has said, ‘sorry, I don’t know why you have this’, and ‘I can’t help you’. It caused me to have more and more anxiety”. Although patients endorsed frustration, stress, or anxiety, some did not blame their providers. These patients felt the issue was one of the broader medical field. They explained that providers are “trying”, but not “enough research has been done” on orofacial pain.

#### 4.2.2. “Drugs, Drugs, Drugs, That’s All They Do”: Disappointed with Ineffective Providers and Care

Patients were often disappointed with the treatment options offered by providers. Patients endorsed the importance of individualized assessments to understand their symptoms and pain, yet rarely received tailored assessment. In fact, some noted that they received care that resulted in worsened pain. Patients expressed that providers overly relied on medications as the first-line treatment, rather than taking a more holistic approach to pain. One participant noted, “All doctors want to do is give you medicine with horrendous side effects with no thought of alternative therapies”. They endorsed negative side effects to medications such as sleepiness, fatigue, and feeling like a “zombie”. Patients also noted that providers rarely offered education and resources for orofacial pain. They felt that many providers did not understand their pain severity and the impact it had on their functioning. They were disappointed when providers did not try to problem-solve after ineffective treatments and concluded they “can’t help” them.

#### 4.2.3. “Some Have Been an Answer to My Prayers”: Appreciative for Effective Providers and Care

Patients also reported positive experiences with providers—although it often took many years to find such a provider. As one participant said, “Went to some who didn’t care but I finally have a good neurologist that listens and does everything he can to help manage everything”. Patients expressed gratitude when providers were knowledgeable about orofacial pain and skillful in planning their care. They endorsed relief and more confidence in their care when providers provide accurate and prompt diagnoses as well as treatment ideas. When patients received effective care, they said it was “life-changing”. Patients also appreciated providers who acknowledged the role of mental health and offered relevant resources.

### 4.3. Domain 3: Patient–Provider Relationships

#### 4.3.1. “They Thought I Was Faking and Drug-Seeking”: Feel Stigmatized and Dismissed by Providers

Patients with orofacial pain felt stigmatized by providers. They noted that some providers “do not take [them] seriously” and suggested the pain was “in [their] head”, which invalidated their experience. Patients were concerned that they were perceived as “faking” pain. Some even felt that their provider “did not want [them] as a patient”. When providers seemed to be “in a rush”, patients felt dismissed. When patients were “diagnosed and dismissed”, they perceived a lack of empathy from providers. One participant explained, “I have had 2/5 that scratched their heads not knowing what to do with me, 2/5 that didn’t believe my pain was a real thing and therefore disregarded me or shoved me off on another doctor, then 1/5 have been doctors that actually know what they are doing and sympathized with me”. Patients said that providers who had poor communication skills made them feel misunderstood and isolated.

#### 4.3.2. “I Hit the Jackpot. He Listens”: Feel Heard and Supported by Providers

Patients also endorsed positive experiences in their relationships with providers. They felt lucky when providers were empathetic, kind, and compassionate. One participant noted, “I have been one of the lucky ones to have doctors who try, listen, and genuinely want to help”. It was important for patients to feel heard. They valued providers who listened, empathized with their challenges and chronic pain, and approached care collaboratively. As one participant expressed, “I’m lucky, my primary care physician, neurologist, and neurosurgeon have always believed me regarding the pain”. Even when providers were unable to treat the pain, patients still appreciated when providers listened to their needs. One participant explained, “All my doctors have been good listeners and highly regarded in the local area. Unfortunately, none have been able to provide any relief to my pain”. Given the complexity of orofacial pain, patients appreciated providers who were accessible and persistent (continued to problem-solve). They felt supported when they believed that providers tried “their best to help”.

### 4.4. Domain 4: Health Systems Factors

#### 4.4.1. “Insurance Has Denied Us Help”: Money, Insurance, and Transportation Are Barriers to Care

Patients highlighted the structural barriers that prevent them from engaging in care. Patients were frustrated with the financial burden of seeing multiple providers and trying exploratory treatments for orofacial pain. New and/or exploratory treatments are costly and rarely supported by insurance companies. As one participant explained, “The problem is my insurance. Everything that the doctors want to try or know will help gets denied because it’s an experimental procedure, because they do not recognize TN [trigeminal neuralgia]. I could list countless treatments and procedures that have been offered to me that have been denied, but they will pay for the tons of medication I take!” At times, patients relied on emergency room visits for acute pain relief when they could not afford consistent outpatient treatment. Patients also endorsed challenges with transportation and logistical barriers to seeing providers. Because of the lack of specialists in orofacial pain, patients often had to travel far distances to receive care, which is challenging for those with limited resources. Because specialists are rare, patients said it was difficult to establish care with a new provider when they moved locations.

#### 4.4.2. “My Current Team Is Not a Team”: Lack of Coordination among Providers

Patients described how challenging it was to coordinate multiple providers. When patients had a team of multidisciplinary providers, they often were not coordinated in their care. One participant explained, “There is never any team management and there is a real disconnect between my GP, neurologist, pain [doctor], and psychologist”. Patients felt as if they were seeking individual consultations and had to streamline their own treatment plan. Patients reported that sometimes they had to manage disagreement among providers and decide which advice to follow when recommendations conflicted. Additionally, some patients were disappointed that their providers did not offer mental health resources. They expressed desire for their teams to be more cohesive and collaborative.

#### 4.4.3. “It Is a Hard Hunt to Find Specialists”: Limited Availability of Orofacial Pain Providers

Patients reported that finding orofacial pain specialists was challenging. They spent many years seeing many different providers—some successful, others unsuccessful. As one participant described, “Finding doctors to, first, believe me, then get a correct diagnosis. Second was the years of trial and error with medications”. Identifying providers who were both knowledgeable and able to treat orofacial pain was challenging and often distressing. For example, a participant said, “Finding a provider knowledgeable about the TMJ is rare. Finding one with TMD knowledge is a unicorn”. The lack of specialized providers created significant emotional distress for patients. One participated shared, “Early on, I may have seen 20 or more doctors that were unable to diagnose my condition, many that were downright rude. This nonsense went on for several years to the point that I no longer wanted to live. I now have a pain doctor that has helped me somewhat get my life back”. Patients highlighted the emotional cost associated with limited resources for their condition including stress, anxiety, depression, and isolation.

## 5. Discussion

Although ~10–15% of adults experience orofacial pain [3], diagnostic assessments and treatments are limited. The search for diagnosis and treatments can be frustrating for both patients and providers. Because of the heterogeneity and subjective experience of orofacial pain, a patient-centered approach is necessary. Positive interactions between patients and providers can improve patient satisfaction and patient-reported outcomes. According to the Patient-Centered Model of Communication [11], patient, provider, relationship, and health system factors can all influence patient–provider interactions. Our study is the first to use this framework to qualitatively explore orofacial pain patients’ experiences with providers. Using a deductive–inductive approach, we identified 10 themes across patient, provider, patient–provider relationship, and health system domains.

Regarding relevant *patient factors*, patients with orofacial pain often felt hopeless about treatments given their history of failed attempts and the lack of understanding from providers about their pain. Patients explained that this hopelessness led to avoidance in continuing to seek care. This aligns with prior literature indicating that patients with orofacial pain often fail to see improvements despite multiple appointments with a variety of providers [17]. Consistent with our findings, these experiences can lead to feelings of hopelessness, resignation, and a lack of faith [8]. Contrary to the prior literature, patients in our study indicated that they developed a sense of agency over their health and needed to take control. They explained that it was important to do their own research and educate medical providers. Prior work indicates that these patients are often perceived as helpless and have a desire to be taken care of by providers [8]. Our findings provide more nuanced and balanced information about patient perspectives—some feel hopeless; others develop agency over their care.

For *provider factors*, patients noted frustration with the lack of provider knowledge about orofacial pain and/or disappointment with ineffective care. Prior work indicates that providers have expressed a similar sentiment of feeling ill-equipped to treat these conditions [6]. Even when diagnosis and treatment are unknown, prior work indicates that strong provider–patient communication can help regulate patient emotions, understand patient needs, and improve patient satisfaction with care [9]. Despite the documented challenges in receiving adequate care [7,18], patients in our study did report positive experiences with providers. They were deeply grateful to providers who helped them find effective care and perceived these treatments as “life-changing”.

Patients’ reports of *patient–provider relationship factors* were both positive and negative. Patients reported feeling stigmatized and dismissed by providers. This finding is consistent with prior research indicating that patients are often met with skepticism, which they perceived as invalidating. Because emotional distress is commonly experienced with orofacial pain [19], it is important that patients feel understood and validated in their pain experience. Extending prior literature, patients also felt heard and supported by providers. They endorsed feeling lucky and grateful when providers were collaborative, listened carefully, and displayed empathy—even if treatments were not successful. The fact that these patients felt lucky suggests that they may expect negative interactions with providers—perhaps because of a history of challenging interactions. Provider–patient relationships are key for patient perceptions of care, outcomes [10], and likely well-being and co-morbid psychosocial distress.

*Health system factors* were an important aspect of patient experiences with providers. Patients reported stress related to insurance coverage, financial burden, and transportation difficulties. They also expressed difficulty finding providers who specialize in orofacial pain and a need for a coordinated multidisciplinary team. These health system factors have rarely been investigated in this population and offer a broader picture of the contextual factors relevant to patient care. Consistent with health disparity research, addressing these systemic factors (e.g., social work support, policy changes) is essential for improving patient health outcomes and psychosocial adjustment to chronic pain [20].

### 5.1. Clinical Implications

Consistent with the Patient-Centered Model of Communication [11], patient, provider, relationship, and health system factors are relevant to patients’ experiences with their providers. Our findings provide a novel and more nuanced understanding of patient–provider interactions. Patients’ experiences with providers vary—they can be negative, positive, and/or neutral. Given this variability, the need for standardized models of patient-centered care is clear. It would be beneficial to develop patient-centered care training opportunities for orofacial pain providers. These could include information on (1) psychosocial factors that impact outcomes; (2) when and how to provide referrals to psychosocial care; (3) communication skills; (4) strategies for working with a multidisciplinary team. Providing psychoeducation on the value of patient–provider interactions for care quality and outcomes could help promote provider buy-in to participate. Our findings also emphasize the need for social work and improved health system policies that can improve access to orofacial pain providers, assessment, and treatments. Improving patient–provider interactions could be a valuable way to enhance patient outcomes, satisfaction with care, and the quality of care delivered. These improvements may help to decrease the high rates of healthcare utilization and costs associated with orofacial pain [21,22].

### 5.2. Strengths and Limitations

This study has several strengths. First, it examined an understudied topic that has the potential to improve patient satisfaction, well-being, and healthcare utilization and costs. Second, we used a hybrid deductive–inductive analytic approach, which allowed us to structure findings using a theoretical framework (Patient-Centered Model of Communication) and identify nuanced themes. Third, we had a considerably large sample of geographically diverse participants compared to most available qualitative studies, which allowed us to capture of a diversity of perspectives. Finally, while much of the research on orofacial pain is siloed within individual diagnoses, we included multiple orofacial pain conditions given their overlapping psychosocial experiences. Understanding experiences of all patients with orofacial pain can help increase the scalability of training initiatives aimed at improving patient–provider communication.

There are important limitations to note. First, our sample was primarily white (79%), which limits our understanding of the experiences of people of color. Future work needs to examine experiences of racially and ethnically diverse participants, especially in relation to health system factors. Second, participants shared their experiences using an open-ended text response. Although this helped us gather a large sample, interview formats would gather additional nuanced information. Finally, we examined experiences of patients and did not include providers. It is important to understand both of their perspectives and integrate that information to capture these interactions.

## 6. Conclusions

Given the complexity of orofacial pain, patient–provider interactions can influence patient reported outcomes, satisfaction with care, and ultimately healthcare utilization and costs. Our findings highlighted the variability in patient experiences—ranging from feeling “diagnosed and dismissed” to feeling that a provider is an “answer to [their] prayers”. These varied experiences emphasize the need for standardized patient-centered care. Training and psychoeducation programs for providers may help to improve understanding of patient experiences, communication, and multidisciplinary team coordination.

## Figures and Tables

**Table 1:** Participant Socio-Demographics.

Variable		*n (%)*
**Gender**		
	Male	33 (12.7%)
	Female	226 (86.9%)
	None stated	1 (0.4%)
**Racial Identity**		
	American Indian or Alaska Native	1 (0.4%)
	Asian or Pacific Islander	1 (0.4%)
	Black or African American	5 (1.9%)
	White	207 (79.6%)
	Multiracial	9 (3.5%)
	None stated	37 (14.2%)
**Ethnicity**		
	Hispanic or Latinx	13 (5.0%)
	Non-Hispanic or Latinx	238 (91.5%)
	None stated	9 (3.5%)
**Diagnostic Category**		
	Trigeminal Neuralgia	130 (50.0%)
	Multiple diagnoses	58 (22.3%)
	TN2	21 (8.1%)
	TNP	18 (6.9%)
	PIFP	14 (5.4%)
	Other	11 (4.2%)
	None stated	8 (3.1%)

***Note*.** TN2 = classical trigeminal neuralgia with concomitant continuous pain (or trigeminal neuralgia type 2 PIFP); TNP = other trigeminal neuropathic pain (or atypical trigeminal neuralgia); PIFP = persistent idiopathic facial pain (or atypical facial pain). “Multiple diagnoses” and “Other” diagnostic categories included: migraines (*n* = 7), myofascial orofacial pain (*n* = 2), temporomandibular disorders (*n* = 6), glossopharyngeal neuralgia (*n* = 10), burning mouth syndrome (*n* = 3), occipital neuralgia (*n* = 6), and diagnoses not specified in the ICOP (*n* = 21).

**Table 2:** Domains, Themes, and Subthemes.

**Domain 1: Patient Factors**	
**Theme**	**Subthemes**
**“It was a failure from the start”:** **Hopeless and avoidant** **about seeking care**	Feel confused and lost in search for care Feel hopeless that “nothing can help” Perception that “there’s nothing left to try” because so many treatments have failed Perception of lack of knowledge and treatment options across the medical community Desire to avoid medical care and providers since “they can’t help me”
**“I needed to take control myself”:** **Initiative and agency needed to** **find appropriate care**	Perception of needing to “educate medical providers” Need to “be responsible” and advocate for appropriate care Need to do own research “to make the right decisions” about treatment
**Domain 2: Provider Factors**	
**“Most don’t have a clue”:** **Frustrated with lack of knowledge about condition and treatment**	Providers “seem mystified” by condition and lack understanding of it Often misdiagnosed because “trying to shove symptoms in a box” Feel they are “bouncing from one doc to the next” to receive diagnosis Perception that providers “are trying but not enough research has been done” It takes a long time to find effective treatments
**“Drugs, drugs, drugs that’s all they do”:** **Disappointed with ineffective** **providers and care**	Often receive ineffective care and/or worsening of pain in response to treatment Providers rarely offer education and individualized assessments Providers overly rely on medication and often lead to negative side effects Providers do not understand pain severity Disappointed when providers do not try to problem-solve and conclude they “can’t help”
**“Some have been an** **answer to my prayers”:** **Appreciative for effective** **providers and care**	Grateful when perceive providers as knowledgeable and skilled Appreciate when providers can make quick, accurate diagnoses Effective treatments “life-changing” even if pain still present Value providers who connect patients with mental health support
**Domain 3: Patient–Provider Relationship Factors**	
**“They thought I was faking** **and drug-seeking”:** **Feel stigmatized and** **dismissed by providers**	Feel stigmatized by providers when they say pain “in your head” Believe they are perceived as “faking pain”, especially in initial visits Report that providers “don’t want me as a patient” Do not have enough time with providers as they are always “in a rush” Lack of trust in providers because “they think I’m making it up” Providers lack empathy as patients feel “diagnosed and dismissed” Providers have poor communication skills
**“I hit the jackpot. He listens”:** **Feel heard and supported by providers**	Felt lucky when providers empathetic, kind, “showed compassion” Grateful when providers are collaborative and listen to patients Value providers who are accessible and persistent (problem-solve) Feel supported when providers determined and “try their best to help”
**Domain 4: Health System Factors**	
**“Insurance has fully** **denied us any help”:** **Money, insurance, and transportation are barriers to care**	Frustration with financial burden and seeing many providers Frustration with inadequate insurance coverage: “insurance is the problem” Rely on emergency room visits for acute pain relief because consistent care is costly Need to travel far distances to seek out specialists Difficult to change providers (e.g., when moving)
**“My current team is not a team”:** **Lack of coordination among providers**	Lack of coordination in multidisciplinary teams interferes with care Lack of support provided for mental health concerns Disagreement among medical professionals is difficult to manage
**“It is a hard hunt to find specialists”:** **Limited availability of** **orofacial pain providers**	Finding a good provider is a “unicorn”, it often takes many years Need to see many different types of providers to find appropriate care

## Data Availability

The data presented in this study are available on request from the corresponding author.
